# 2,3-Dicyano-4-[(4-methyl­phenyl­sulfon­yl)­oxy]phenyl 4-methyl­benzene­sulfonate

**DOI:** 10.1107/S1600536811009160

**Published:** 2011-03-15

**Authors:** Yanhua Deng, Changqin Ma, Xiaomei Zhang

**Affiliations:** a School of Chemistry and Chemical Technology, Shandong University, Jinan 250100, People’s Republic of China

## Abstract

In the title compound, C_22_H_16_N_2_O_6_S_2_, the dihedral angle formed by the mean planes of the two benzene rings of the 4-methyl­phenyl­sulfonate groups is 21.9 (1)° and these rings form dihedral angles of 48.26 (9) and 52.73 (9)° with the central benzene ring.

## Related literature

For the applications of phthalocyanines, see: Kobayashi (2001 ▶); Shirk & Pong (2000 ▶); Lukyanets (1999 ▶). For the synthetic procedure, see: Rey *et al.* (1998 ▶). For a related structure, see: Zhang *et al.* (2009 ▶). For standard bond distances, see: Allen *et al.* (1987 ▶).
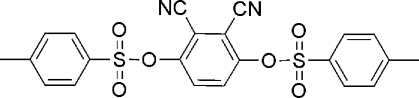


## Experimental

### 

#### Crystal data

C_22_H_16_N_2_O_6_S_2_
*M* *_r_* = 468.49
Monoclinic, 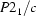

*a* = 6.2484 (16) Å
*b* = 21.478 (6) Å
*c* = 16.331 (4) Å
β = 94.940 (4)°
*V* = 2183.5 (10) Å^3^
*Z* = 4
Mo *K*α radiation
μ = 0.29 mm^−1^
*T* = 293 K
0.42 × 0.31 × 0.26 mm

#### Data collection

Bruker SMART CCD area-detector diffractometer
Absorption correction: multi-scan (*SADABS*; Sheldrick, 1996 ▶) *T*_min_ = 0.889, *T*_max_ = 0.929
10754 measured reflections
3848 independent reflections
3237 reflections with *I* > 2σ(*I*)
*R*_int_ = 0.021

#### Refinement

*R*[*F* ^2^ > 2σ(*F* ^2^)] = 0.034
*wR*(*F* ^2^) = 0.098
*S* = 1.03
3848 reflections
291 parameters
H-atom parameters constrained
Δρ_max_ = 0.22 e Å^−3^
Δρ_min_ = −0.28 e Å^−3^

### 

Data collection: *SMART* (Bruker, 1997 ▶); cell refinement: *SAINT* (Bruker, 1997 ▶); data reduction: *SAINT*; program(s) used to solve structure: *SHELXS97* (Sheldrick, 2008 ▶); program(s) used to refine structure: *SHELXL97* (Sheldrick, 2008 ▶); molecular graphics: *DIAMOND* (Brandenburg, 1999 ▶); software used to prepare material for publication: *SHELXTL* (Sheldrick, 2008 ▶).

## Supplementary Material

Crystal structure: contains datablocks global, I. DOI: 10.1107/S1600536811009160/lh5215sup1.cif

Structure factors: contains datablocks I. DOI: 10.1107/S1600536811009160/lh5215Isup2.hkl

Additional supplementary materials:  crystallographic information; 3D view; checkCIF report

## Figures and Tables

**Fig. 1.**
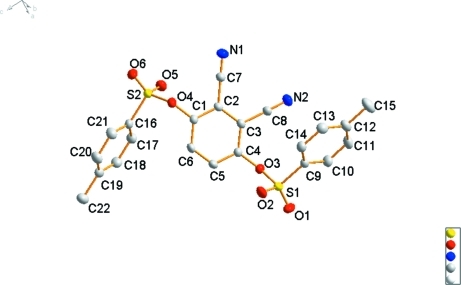
A view of the title compound with displacement ellipsoids drawn at the 30% probability level.

## supplementary crystallographic information

### Comment

Dicyano compounds have been widely used to synthesize many useful materials such as phthalocyanines. Phthalocyanines are an interesting class of compounds, with increasingly diverse industrial and biomedical applications, for instance as liquid crystals, materials for optical storage (Kobayashi, 2001), oxidation catalysts, solar cell functional materials, gas sensors, nonlinear optical limiting devices (Shirk & Pong, 2000), photodynamic therapy agents (Lukyanets, 1999) and phthalocyanine dyes (Zhang *et al.* 2009).

The crystal structure of the title compound is shown in Fig. 1. The dihedral angle formed by the the mean planes of the two benzene rings of the 4-methylphenylsulfonate groups is 21.9 (1)° and each of these rings forms didhedral angles of 48.26 (9)° [C9-C14] and 52.73 (9)° [C16-C21] with the central benzene ring [C1-C6]. The bond distances (Allen *et al.* 1987) and angles are as expected and similar to those which are related in 4,5-biaminobenzene-1,2-dicarbonitrile (Zhang *et al.*, 2009).

### Experimental

The title compound was prepared according to the method of Rey *et al.* (1998).

### Refinement

Hydrogen atoms were placed in calculated positions and refined using a riding-model approximation with C—H = 0.93 Å, *U*_iso_ = 1.2*U*_eq_ (C) for aromatic H atoms and C—H = 0.96 Å, *U*_iso_ = 1.5*U*_eq_ (C) for methyl H atoms.

### Crystal data

**Table:**

C_22_H_16_N_2_O_6_S_2_	*Z* = 4
*M**_r_* = 468.49	*F*(000) = 968
Monoclinic, *P*2_1_/*c*	*D*_x_ = 1.425 Mg m^−^^3^
Hall symbol: -P 2ybc	Mo *K*α radiation, λ = 0.71073 Å
*a* = 6.2484 (16) Å	Cell parameters from 3848 reflections
*b* = 21.478 (6) Å	µ = 0.29 mm^−^^1^
*c* = 16.331 (4) Å	*T* = 293 K
β = 94.940 (4)°	Block, colorless
*V* = 2183.5 (10) Å^3^	0.42 × 0.31 × 0.26 mm

### Data collection

**Table:**

Bruker SMART CCD area-detector diffractometer	3848 independent reflections
Radiation source: fine-focus sealed tube	3237 reflections with *I* > 2σ(*I*)
graphite	*R*_int_ = 0.021
φ and ω scans	θ_max_ = 25.0°, θ_min_ = 1.6°
Absorption correction: multi-scan (*SADABS*; Sheldrick, 1996)	*h* = −7→7
*T*_min_ = 0.889, *T*_max_ = 0.929	*k* = −25→23
10754 measured reflections	*l* = −19→17

### Refinement

**Table:**

Refinement on *F*^2^	Primary atom site location: structure-invariant direct methods
Least-squares matrix: full	Secondary atom site location: difference Fourier map
*R*[*F*^2^ > 2σ(*F*^2^)] = 0.034	Hydrogen site location: inferred from neighbouring sites
*wR*(*F*^2^) = 0.098	H-atom parameters constrained
*S* = 1.03	*w* = 1/[σ^2^(*F*_o_^2^) + (0.0498*P*)^2^ + 0.6924*P*] where *P* = (*F*_o_^2^ + 2*F*_c_^2^)/3
3848 reflections	(Δ/σ)_max_ = 0.018
291 parameters	Δρ_max_ = 0.22 e Å^−^^3^
0 restraints	Δρ_min_ = −0.28 e Å^−^^3^

### Special details

**Table:**

Geometry. All e.s.d.'s (except the e.s.d. in the dihedral angle between two l.s. planes) are estimated using the full covariance matrix. The cell e.s.d.'s are taken into account individually in the estimation of e.s.d.'s in distances, angles and torsion angles; correlations between e.s.d.'s in cell parameters are only used when they are defined by crystal symmetry. An approximate (isotropic) treatment of cell e.s.d.'s is used for estimating e.s.d.'s involving l.s. planes.
Refinement. Refinement of *F*^2^ against ALL reflections. The weighted *R*-factor *wR* and goodness of fit *S* are based on *F*^2^, conventional *R*-factors *R* are based on *F*, with *F* set to zero for negative *F*^2^. The threshold expression of *F*^2^ > σ(*F*^2^) is used only for calculating *R*-factors(gt) *etc*. and is not relevant to the choice of reflections for refinement. *R*-factors based on *F*^2^ are statistically about twice as large as those based on *F*, and *R*- factors based on ALL data will be even larger.

### Fractional atomic coordinates and isotropic or equivalent isotropic displacement parameters (Å^2^)

**Table:**

	*x*	*y*	*z*	*U*_iso_*/*U*_eq_	
S1	1.13975 (8)	0.52784 (2)	0.81677 (3)	0.04334 (16)	
S2	0.32466 (8)	0.28406 (2)	0.95047 (3)	0.04432 (16)	
O1	1.3671 (2)	0.52925 (8)	0.82002 (10)	0.0664 (5)	
O2	1.0278 (3)	0.55562 (7)	0.87978 (8)	0.0590 (4)	
O3	1.0886 (2)	0.45361 (6)	0.81606 (8)	0.0403 (3)	
O4	0.33997 (19)	0.35694 (6)	0.92363 (8)	0.0433 (3)	
O5	0.4229 (2)	0.24810 (7)	0.89114 (9)	0.0565 (4)	
O6	0.1032 (2)	0.27798 (8)	0.96165 (9)	0.0639 (5)	
N1	0.2599 (3)	0.33703 (9)	0.70932 (11)	0.0598 (5)	
N2	0.7716 (3)	0.41758 (10)	0.62978 (11)	0.0648 (6)	
C1	0.5326 (3)	0.37925 (9)	0.89697 (11)	0.0364 (4)	
C2	0.5550 (3)	0.38090 (8)	0.81323 (10)	0.0345 (4)	
C3	0.7409 (3)	0.40812 (8)	0.78578 (10)	0.0346 (4)	
C4	0.8981 (3)	0.43099 (8)	0.84335 (11)	0.0354 (4)	
C5	0.8736 (3)	0.42786 (9)	0.92640 (11)	0.0411 (4)	
H5	0.9816	0.4427	0.9642	0.049*	
C6	0.6896 (3)	0.40281 (9)	0.95348 (11)	0.0419 (5)	
H6	0.6709	0.4017	1.0093	0.050*	
C7	0.3899 (3)	0.35619 (9)	0.75541 (11)	0.0405 (4)	
C8	0.7631 (3)	0.41295 (9)	0.69862 (11)	0.0411 (4)	
C9	1.0274 (3)	0.55195 (8)	0.72064 (11)	0.0380 (4)	
C10	1.1458 (3)	0.54618 (10)	0.65330 (12)	0.0495 (5)	
H10	1.2845	0.5301	0.6592	0.059*	
C11	1.0549 (4)	0.56462 (11)	0.57751 (13)	0.0585 (6)	
H11	1.1342	0.5611	0.5321	0.070*	
C12	0.8488 (4)	0.58828 (10)	0.56718 (13)	0.0527 (5)	
C13	0.7331 (4)	0.59329 (10)	0.63583 (13)	0.0520 (5)	
H13	0.5942	0.6091	0.6298	0.062*	
C14	0.8195 (3)	0.57529 (9)	0.71285 (12)	0.0440 (5)	
H14	0.7405	0.5787	0.7584	0.053*	
C15	0.7522 (5)	0.60828 (14)	0.48352 (15)	0.0837 (9)	
H15A	0.8225	0.6454	0.4671	0.126*	
H15B	0.6018	0.6164	0.4858	0.126*	
H15C	0.7710	0.5758	0.4444	0.126*	
C16	0.4794 (3)	0.28074 (9)	1.04487 (11)	0.0389 (4)	
C17	0.6793 (3)	0.25268 (10)	1.04910 (13)	0.0481 (5)	
H17	0.7307	0.2355	1.0023	0.058*	
C18	0.8014 (3)	0.25053 (10)	1.12350 (13)	0.0512 (5)	
H18	0.9358	0.2317	1.1266	0.061*	
C19	0.7272 (3)	0.27601 (9)	1.19388 (12)	0.0446 (5)	
C20	0.5257 (4)	0.30354 (10)	1.18779 (12)	0.0527 (5)	
H20	0.4736	0.3206	1.2345	0.063*	
C21	0.4005 (3)	0.30622 (10)	1.11400 (12)	0.0491 (5)	
H21	0.2657	0.3248	1.1108	0.059*	
C22	0.8603 (4)	0.27260 (12)	1.27527 (14)	0.0618 (6)	
H22A	0.8330	0.3087	1.3075	0.093*	
H22B	1.0099	0.2712	1.2660	0.093*	
H22C	0.8227	0.2358	1.3041	0.093*	

### Atomic displacement parameters (Å^2^)

**Table:**

	*U*^11^	*U*^22^	*U*^33^	*U*^12^	*U*^13^	*U*^23^
S1	0.0492 (3)	0.0430 (3)	0.0363 (3)	−0.0115 (2)	−0.0055 (2)	0.0044 (2)
S2	0.0403 (3)	0.0553 (3)	0.0370 (3)	−0.0110 (2)	0.0015 (2)	0.0056 (2)
O1	0.0470 (9)	0.0735 (11)	0.0752 (11)	−0.0216 (8)	−0.0154 (8)	0.0208 (8)
O2	0.0942 (12)	0.0491 (9)	0.0331 (8)	−0.0083 (8)	0.0020 (7)	−0.0044 (6)
O3	0.0385 (7)	0.0393 (7)	0.0430 (7)	−0.0032 (5)	0.0033 (6)	0.0055 (6)
O4	0.0350 (7)	0.0556 (8)	0.0396 (7)	−0.0001 (6)	0.0038 (6)	0.0105 (6)
O5	0.0697 (10)	0.0563 (9)	0.0436 (8)	−0.0083 (7)	0.0052 (7)	−0.0055 (7)
O6	0.0414 (8)	0.0942 (13)	0.0556 (9)	−0.0221 (8)	0.0014 (7)	0.0152 (8)
N1	0.0575 (12)	0.0685 (13)	0.0504 (11)	−0.0097 (10)	−0.0119 (9)	−0.0008 (9)
N2	0.0778 (14)	0.0821 (14)	0.0347 (11)	−0.0164 (11)	0.0057 (9)	0.0001 (9)
C1	0.0366 (10)	0.0391 (10)	0.0331 (10)	−0.0001 (8)	0.0011 (8)	0.0066 (8)
C2	0.0362 (10)	0.0362 (10)	0.0302 (9)	0.0023 (8)	−0.0026 (7)	0.0026 (7)
C3	0.0413 (10)	0.0335 (9)	0.0287 (9)	0.0015 (8)	0.0011 (8)	0.0024 (7)
C4	0.0380 (10)	0.0341 (9)	0.0339 (10)	−0.0021 (8)	0.0015 (8)	0.0048 (7)
C5	0.0462 (11)	0.0443 (11)	0.0310 (10)	−0.0069 (9)	−0.0067 (8)	0.0038 (8)
C6	0.0493 (12)	0.0502 (11)	0.0258 (9)	−0.0044 (9)	0.0014 (8)	0.0047 (8)
C7	0.0409 (11)	0.0460 (11)	0.0339 (10)	−0.0020 (9)	−0.0007 (8)	0.0031 (8)
C8	0.0447 (11)	0.0455 (11)	0.0326 (11)	−0.0052 (9)	0.0008 (8)	−0.0005 (8)
C9	0.0446 (11)	0.0363 (10)	0.0330 (10)	−0.0047 (8)	0.0025 (8)	0.0025 (8)
C10	0.0468 (12)	0.0575 (13)	0.0453 (12)	0.0033 (10)	0.0101 (9)	0.0053 (10)
C11	0.0711 (16)	0.0677 (15)	0.0386 (12)	0.0055 (12)	0.0165 (11)	0.0062 (10)
C12	0.0712 (15)	0.0490 (12)	0.0367 (11)	0.0026 (11)	−0.0028 (10)	0.0027 (9)
C13	0.0516 (13)	0.0485 (12)	0.0546 (13)	0.0065 (10)	−0.0033 (10)	0.0034 (10)
C14	0.0475 (12)	0.0456 (11)	0.0398 (11)	0.0019 (9)	0.0089 (9)	0.0021 (9)
C15	0.115 (2)	0.088 (2)	0.0452 (14)	0.0199 (17)	−0.0107 (14)	0.0095 (13)
C16	0.0400 (10)	0.0415 (10)	0.0355 (10)	−0.0049 (8)	0.0047 (8)	0.0084 (8)
C17	0.0434 (11)	0.0595 (13)	0.0423 (11)	0.0019 (10)	0.0085 (9)	0.0015 (10)
C18	0.0387 (11)	0.0584 (13)	0.0558 (13)	0.0042 (9)	0.0005 (10)	0.0078 (10)
C19	0.0485 (12)	0.0408 (11)	0.0436 (11)	−0.0064 (9)	−0.0019 (9)	0.0091 (9)
C20	0.0623 (14)	0.0578 (13)	0.0381 (11)	0.0120 (11)	0.0041 (10)	−0.0009 (10)
C21	0.0454 (12)	0.0556 (13)	0.0465 (12)	0.0130 (10)	0.0044 (9)	0.0039 (10)
C22	0.0658 (15)	0.0642 (15)	0.0523 (13)	−0.0046 (12)	−0.0126 (11)	0.0068 (11)

### Geometric parameters (Å, °)

**Table:**

S1—O1	1.4176 (16)	C10—H10	0.9300
S1—O2	1.4240 (16)	C11—C12	1.381 (3)
S1—O3	1.6259 (14)	C11—H11	0.9300
S1—C9	1.7422 (19)	C12—C13	1.389 (3)
S2—O6	1.4173 (15)	C12—C15	1.508 (3)
S2—O5	1.4193 (16)	C13—C14	1.381 (3)
S2—O4	1.6303 (15)	C13—H13	0.9300
S2—C16	1.7493 (19)	C14—H14	0.9300
O3—C4	1.394 (2)	C15—H15A	0.9600
O4—C1	1.399 (2)	C15—H15B	0.9600
N1—C7	1.136 (2)	C15—H15C	0.9600
N2—C8	1.135 (2)	C16—C21	1.383 (3)
C1—C6	1.383 (3)	C16—C17	1.383 (3)
C1—C2	1.387 (2)	C17—C18	1.379 (3)
C2—C3	1.408 (2)	C17—H17	0.9300
C2—C7	1.439 (3)	C18—C19	1.388 (3)
C3—C4	1.390 (2)	C18—H18	0.9300
C3—C8	1.446 (2)	C19—C20	1.387 (3)
C4—C5	1.380 (2)	C19—C22	1.508 (3)
C5—C6	1.377 (3)	C20—C21	1.380 (3)
C5—H5	0.9300	C20—H20	0.9300
C6—H6	0.9300	C21—H21	0.9300
C9—C10	1.383 (3)	C22—H22A	0.9600
C9—C14	1.388 (3)	C22—H22B	0.9600
C10—C11	1.375 (3)	C22—H22C	0.9600
			
O1—S1—O2	121.13 (10)	C10—C11—H11	119.2
O1—S1—O3	102.52 (8)	C12—C11—H11	119.2
O2—S1—O3	107.91 (8)	C11—C12—C13	118.31 (19)
O1—S1—C9	110.63 (9)	C11—C12—C15	120.8 (2)
O2—S1—C9	109.99 (9)	C13—C12—C15	120.9 (2)
O3—S1—C9	102.78 (8)	C14—C13—C12	121.6 (2)
O6—S2—O5	121.48 (10)	C14—C13—H13	119.2
O6—S2—O4	101.75 (9)	C12—C13—H13	119.2
O5—S2—O4	107.47 (8)	C13—C14—C9	118.30 (18)
O6—S2—C16	110.75 (9)	C13—C14—H14	120.8
O5—S2—C16	109.93 (10)	C9—C14—H14	120.8
O4—S2—C16	103.61 (8)	C12—C15—H15A	109.5
C4—O3—S1	120.81 (11)	C12—C15—H15B	109.5
C1—O4—S2	118.97 (11)	H15A—C15—H15B	109.5
C6—C1—C2	121.50 (17)	C12—C15—H15C	109.5
C6—C1—O4	119.80 (16)	H15A—C15—H15C	109.5
C2—C1—O4	118.56 (16)	H15B—C15—H15C	109.5
C1—C2—C3	118.73 (16)	C21—C16—C17	121.02 (18)
C1—C2—C7	120.66 (17)	C21—C16—S2	119.64 (15)
C3—C2—C7	120.60 (16)	C17—C16—S2	119.34 (15)
C4—C3—C2	119.08 (16)	C18—C17—C16	119.17 (19)
C4—C3—C8	121.24 (16)	C18—C17—H17	120.4
C2—C3—C8	119.67 (16)	C16—C17—H17	120.4
C5—C4—C3	121.06 (17)	C17—C18—C19	121.17 (19)
C5—C4—O3	120.10 (16)	C17—C18—H18	119.4
C3—C4—O3	118.70 (15)	C19—C18—H18	119.4
C6—C5—C4	120.09 (17)	C20—C19—C18	118.32 (18)
C6—C5—H5	120.0	C20—C19—C22	120.83 (19)
C4—C5—H5	120.0	C18—C19—C22	120.83 (19)
C5—C6—C1	119.51 (17)	C21—C20—C19	121.53 (19)
C5—C6—H6	120.2	C21—C20—H20	119.2
C1—C6—H6	120.2	C19—C20—H20	119.2
N1—C7—C2	179.5 (2)	C20—C21—C16	118.78 (19)
N2—C8—C3	177.1 (2)	C20—C21—H21	120.6
C10—C9—C14	121.33 (18)	C16—C21—H21	120.6
C10—C9—S1	119.23 (15)	C19—C22—H22A	109.5
C14—C9—S1	119.42 (14)	C19—C22—H22B	109.5
C11—C10—C9	118.8 (2)	H22A—C22—H22B	109.5
C11—C10—H10	120.6	C19—C22—H22C	109.5
C9—C10—H10	120.6	H22A—C22—H22C	109.5
C10—C11—C12	121.6 (2)	H22B—C22—H22C	109.5
			
O1—S1—O3—C4	155.86 (13)	O2—S1—C9—C10	160.90 (16)
O2—S1—O3—C4	26.93 (15)	O3—S1—C9—C10	−84.39 (17)
C9—S1—O3—C4	−89.28 (14)	O1—S1—C9—C14	−156.90 (16)
O6—S2—O4—C1	−172.14 (13)	O2—S1—C9—C14	−20.45 (19)
O5—S2—O4—C1	−43.49 (15)	O3—S1—C9—C14	94.26 (16)
C16—S2—O4—C1	72.86 (14)	C14—C9—C10—C11	0.6 (3)
S2—O4—C1—C6	−88.93 (19)	S1—C9—C10—C11	179.22 (17)
S2—O4—C1—C2	95.31 (17)	C9—C10—C11—C12	−0.4 (3)
C6—C1—C2—C3	−1.0 (3)	C10—C11—C12—C13	0.1 (4)
O4—C1—C2—C3	174.72 (16)	C10—C11—C12—C15	179.9 (2)
C6—C1—C2—C7	−179.95 (18)	C11—C12—C13—C14	0.0 (3)
O4—C1—C2—C7	−4.3 (3)	C15—C12—C13—C14	−179.9 (2)
C1—C2—C3—C4	1.6 (3)	C12—C13—C14—C9	0.2 (3)
C7—C2—C3—C4	−179.39 (17)	C10—C9—C14—C13	−0.5 (3)
C1—C2—C3—C8	−177.15 (17)	S1—C9—C14—C13	−179.14 (15)
C7—C2—C3—C8	1.8 (3)	O6—S2—C16—C21	−35.2 (2)
C2—C3—C4—C5	−0.6 (3)	O5—S2—C16—C21	−172.16 (16)
C8—C3—C4—C5	178.17 (17)	O4—S2—C16—C21	73.23 (17)
C2—C3—C4—O3	175.24 (15)	O6—S2—C16—C17	144.94 (17)
C8—C3—C4—O3	−6.0 (3)	O5—S2—C16—C17	7.95 (19)
S1—O3—C4—C5	−76.16 (19)	O4—S2—C16—C17	−106.66 (16)
S1—O3—C4—C3	107.97 (17)	C21—C16—C17—C18	−0.3 (3)
C3—C4—C5—C6	−1.2 (3)	S2—C16—C17—C18	179.56 (16)
O3—C4—C5—C6	−176.94 (17)	C16—C17—C18—C19	0.0 (3)
C4—C5—C6—C1	1.8 (3)	C17—C18—C19—C20	0.4 (3)
C2—C1—C6—C5	−0.8 (3)	C17—C18—C19—C22	179.1 (2)
O4—C1—C6—C5	−176.40 (17)	C18—C19—C20—C21	−0.3 (3)
C1—C2—C7—N1	149 (100)	C22—C19—C20—C21	−179.0 (2)
C3—C2—C7—N1	−30 (31)	C19—C20—C21—C16	0.0 (3)
C4—C3—C8—N2	−134 (4)	C17—C16—C21—C20	0.4 (3)
C2—C3—C8—N2	44 (4)	S2—C16—C21—C20	−179.53 (16)
O1—S1—C9—C10	24.45 (19)		
